# Detection of 10 cases of ceftriaxone-resistant *Neisseria gonorrhoeae* in the United Kingdom, December 2021 to June 2022

**DOI:** 10.2807/1560-7917.ES.2022.27.46.2200803

**Published:** 2022-11-17

**Authors:** Michaela Day, Rachel Pitt, Nisha Mody, John Saunders, Rupa Rai, Achyuta Nori, Hannah Church, Sarah Mensforth, Helen Corkin, Jacqueline Jones, Preneshni Naicker, Wazirzada M Khan, Rebecca Thomson Glover, Kalani Mortimer, Chloe Hylton, Elizabeth Moss, Thomas Joshua Pasvol, Ania Richardson, Suzy Sun, Neil Woodford, Hamish Mohammed, Katy Sinka, Helen Fifer

**Affiliations:** 1 National Incident Management Team, United Kingdom Health Security Agency, London, United Kingdom

**Keywords:** gonorrhoea, resistance

## Abstract

Between December 2021 and June 2022, 10 cases of ceftriaxone-resistant *Neisseria gonorrhoeae* (ST8123; n = 8) were detected in the United Kingdom, compared with nine cases during the previous 6 years. Most of these cases were associated with travel from the Asia-Pacific region; all were heterosexual people, with most in their 20s. Although all cases were successfully treated, not all partners of cases could be traced, and there is a risk of further transmission of ceftriaxone-resistant gonococcal infection within the UK.

*Neisseria gonorrhoeae* has developed resistance to all antibiotics recommended for treatment, including ceftriaxone, the last-line option for empirical monotherapy. The *N. gonorrhoeae* FC428 clone, which is associated with ceftriaxone-resistance, has been detected in numerous countries, usually with epidemiological links to countries in the Asia-Pacific region including China, Japan, the Philippines, Thailand and Vietnam [1-6]. Between December 2015 and September 2021, nine cases of ceftriaxone-resistant *N. gonorrhoeae* were detected in the United Kingdom (UK), all associated with international travel. We report 10 cases of ceftriaxone-resistant *N. gonorrhoeae* presenting to sexual health services (SHSs) across the UK in just 6 months, between December 2021 and June 2022.

## Case descriptions

Eight cases were heterosexual people (four men and four women) in their 20s from the same Asia-Pacific country. The majority had travelled to different areas of the UK to study. These eight comprised three partnerships and two individuals. In two partnerships, infection was acquired in the Asia-Pacific region before UK arrival. In the remaining cases, infection was acquired in the UK from sex with one-off uncontactable partners who were nationals of the same country. All four men presented with urethral discharge. Three of the women presented as asymptomatic contacts of their male partners. The fourth woman presented to an eye clinic with conjunctivitis and was advised to attend a SHS for STI screening, whereupon she was diagnosed with asymptomatic genital gonorrhoea (conjunctival *N. gonorrhoeae* nucleic acid amplification test (NAAT) and culture negative).

Case 9 was a woman in her 20s who had a partner of 2 months duration, her only partner since a negative sexually transmitted infection (STI) screen 6 months previously. Neither had travelled or had epidemiological links to the other cases. Following the diagnosis in Case 9, the partner had two negative urine NAATs for *N. gonorrhoeae*.

These nine cases had positive genital *N. gonorrhoeae* NAATs and cultures (Table). Seven were treated empirically with ceftriaxone 1 g intramuscularly (IM). Two received azithromycin 2 g orally. All had negative genital and pharyngeal *N. gonorrhoeae* NAATs and cultures at test-of-cure taken between 2–3 weeks after treatment.

**Table:** Phenotypic susceptibility and typing results for 10 ceftriaxone-resistant *N. gonorrhoeae* isolates detected in the United Kingdom, December 2021–June 2022

Typing and susceptibility		Case									
		1	2	3	4	5	6	7	8	9	10
MLST ST		8123	8123	8123	8123	8123	8123	8123	8123	1901	16406
NG-STAR profile		60.001_22_8_1_34_93_100	60.001_22_8_1_34_93_100	60.001_22_8_1_34_93_100	60.001_22_8_1_34_93_2	60.001_22_8_1_34_93_2	60.001_22_8_1_34_24_100	60.001_22_8_1_34_24_2	60.001_22_8_1_34_24_100	237.001_1_8_1_7_3_100	60.001_89_13_1_1_176_1
23S rRNA		wt	wt	wt	C2611T	C2611T	wt	C2611T	wt	wt	A2059G
*penA*		60.001	60.001	60.001	60.001	60.001	60.001	60.001	60.001	237.001	60.001
Ceftriaxone	MIC, mg/L	0.25	1	1	0.5	0.5	0.5	0.25	0.5	1	0.25
	SIR	R	R	R	R	R	R	R	R	R	R
Cefixime	MIC, mg/L	0.5	1	1	1	1	1	1	1	2	1
	SIR	R	R	R	R	R	R	R	R	R	R
Azithromycin	MIC, mg/L	0.25	0.5	0.25	64	64	0.5	16	1	0.25	> 256
	SIR^a^	S	S	S	R	R	S	R	S	S	R
Ciprofloxacin	MIC, mg/L	16	> 32	> 32	> 32	> 32	> 32	> 32	> 32	32	8
	SIR	R	R	R	R	R	R	R	R	R	R
Penicillin	MIC, mg/L	16	> 32	> 32	16	32	16	16	16	2	> 32
	SIR	R	R	R	R	R	R	R	R	R	R
Tetracycline	MIC, mg/L	0.25	1	1	2	1	1	0.5	1	1	8
	SIR	S	I	I	R	I	I	S	I	I	R
Spectinomycin	MIC, mg/L	4	8	8	16	8	8	8	8	8	8
	SIR	S	S	S	S	S	S	S	S	S	S
Gentamicin	MIC, mg/L	2	4	4	4	4	4	4	8	8	2
Ertapenem	MIC, mg/L	0.008	0.016	0.016	0.016	0.016	0.032	0.016	0.032	0.064	0.016

I: susceptible, increased exposure; MIC: minimum inhibitory concentration; MLST ST: multilocus sequence typing sequence type; NG-STAR: *N. gonorrhoeae* sequence typing for antimicrobial resistance; R: resistant; S: susceptible, standard dosing regimen; SIR: susceptibility category; wt: wild-type.

^a^ No EUCAST breakpoint for azithromycin. MICs greater than the epidemiological cut off value (1 mg/L) are represented as R, and below the cut off value as S.

Case 10 was a heterosexual man in his 40s reporting condomless vaginal sex with an uncontactable female resident of another Asia-Pacific country while visiting the region. He developed urethral discharge and was initially treated with oral cefixime and azithromycin (dosage not known) but his symptoms persisted. On return to the UK, he received ceftriaxone 1 g IM. *N. gonorrhoeae* was cultured from urethral and pharyngeal swabs (Table). Cultures and NAATs were negative 2 weeks later. He reported not having had oral sex while in the Asia-Pacific region or with any sexual partners in the UK.

## Characterisation of *Neisseria gonorrhoeae* isolates

All isolates were sent to the UK Health Security Agency (UKHSA) reference laboratory where *N. gonorrhoeae* was confirmed by MALDI-TOF (Bruker) and minimum inhibitory concentrations (MICs) determined using Etest (BioMerieux) on GC agar (BD) supplemented with 1% Vitox (Oxoid). MICs were interpreted with EUCAST clinical breakpoints (Table) [7].

Genomic DNA was extracted using the Qiasymphony SP using the DSP DNA mini kit (Qiagen). Illumina sequencing was performed and raw fastq files assembled using SPAdes (Galaxy v3.5.0) and uploaded to PathogenWatch [8] for typing and resistance gene detection and variant calling. A phylogenetic tree was generated in PathogenWatch using pairwise distance scores based on the number of different loci (excluding any missing loci) for the core genome multilocus sequence typing (cgMLST) [8]. Isolates from Cases 1–8 were sequence type (ST) 8123, whereas the isolates from Cases 9 and 10 were different STs (Figure).

**Figure:**
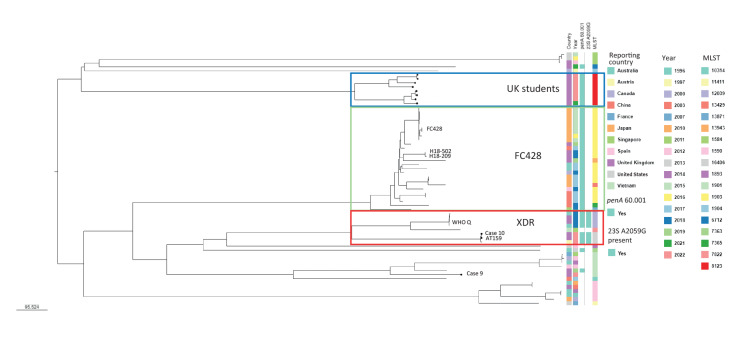
Phylogeny of recent United Kingdom ceftriaxone-resistant *Neisseria gonorrhoeae* genome sequences (n = 10) with all predicted ceftriaxone-resistant *N. gonorrhoeae* genome sequences (n = 52) publicly available in PathogenWatch, up to July 2022

MLST: multilocus sequence typing; UK: United Kingdom; XDR: extensively drug-resistant.

Phylogeny based on cgMLST was generated using PathogenWatch [8]. The 10 UK ceftriaxone-resistant cases described in this article are represented by black circles. Case 10 has two identical isolates from two different anatomical sites. Cases 1–8 are shown in the blue box. The green box shows the original FC428 clone characterised in Japan in 2015 [4] including two UK cases linked to travel to Ibiza in 2018, H18-502 and H18-209 [20]. The red box shows XDR isolates including WHO Q, a UK case linked to travel to Thailand in 2018 [5], AT159, an Austrian case linked to travel to Cambodia in April 2022 [6], and Case 10. The 23S rRNA A2059G mutation in three or four alleles confers high-level resistance to azithromycin (MIC > 256 mg/L).

Please note all comparator strains in the tree have predicted ceftriaxone resistance from whole genome sequencing data but this has not been confirmed phenotypically for all strains shown (either not tested/unpublished or MIC < 0.125 mg/L) including the two UK strains from 2003 (MIC: 0.064 mg/L) and 2014 (MIC data unpublished).

Ceftriaxone resistance resulted from a mosaic *penA*-60.001 allele in nine isolates and a mosaic *penA*-237.001 allele for one isolate (from Case 9). Azithromycin MICs for six isolates were low (≤ 1 mg/L), however, three isolates (MICs 16–64 mg/L) had the C2611T mutation in their 23S rRNA genes and one had a 23S rRNA A2509G mutation (MIC > 256 mg/L) (Table).

## Discussion

The eight ST8123 *N. gonorrhoeae* isolates we describe are currently the only examples of ceftriaxone-resistant ST8123 strains within the PathogenWatch database. The ST8123 lineage has not been widely reported within Europe, with three isolates identified in the Euro-GASP 2013 study [9] and two in the Euro-GASP 2018 study [10]. Within PathogenWatch, there are an additional 12 isolates reported from Vietnam, the UK, Guinea-Bissau and the United States [8]. However, there are reports of this being the most common ST in Shenzhen, China among isolates collected between 2014 and 2018 [11]. No WGS data were available from that study.

The isolate from Case 7 was almost identical to the isolate from Case 8 (difference in 23S rRNA C2611T mutation) although these cases were not known to be partners. These two isolates were collected within 2 weeks of each other and in the same UK region. The presence of the 23S rRNA C2611T mutation in the isolate from Case 7 but not in the isolate from Case 8 could suggest that the 23S rRNA mutation occurred within Case 7. Reversion of 23S rRNA C2611T to wild-type has been demonstrated in vitro so this also cannot be ruled out [12].

The isolate from Case 10 (ST16406) was identical to the recently reported AT159 ceftriaxone-resistant strain linked with travel to Cambodia [6]. Both are also related to strain WHO Q from a case reported in the UK in 2018 with travel links to Thailand [5]. All of these isolates also had high-level resistance to azithromycin (MIC > 256 mg/L) and therefore were classified as extensively-drug resistant [5]. The similarity of the three strains, along with the travel links, strongly suggest these strains are circulating within the Asia-Pacific region.

The isolate from Case 9, ST1901, is distinct from the other nine cases and is most closely related to a ceftriaxone-resistant isolate from a male from the Asia-Pacific detected in the UK in 2019. Sequence type 1901 is internationally disseminated and has been the prevalent ceftriaxone-resistant clone in Shenzhen, China since 2014 [11], with high prevalence also reported in South Korea [13], Argentina [14] and Japan [15] and with sporadic reports associated with treatment failures reported across Europe [16-18].

For some of the cases reported here, the source of infection was not clear and not all partners could be contacted, therefore there is a risk of ongoing transmission within the UK. It is reassuring that all cases had a negative test-of-cure. Pharyngeal infection is more difficult to treat than genital infection and treatment failures have previously been documented for pharyngeal infections [5]. Only one of the 10 cases reported here was known to have had a pharyngeal infection, and this also cleared following treatment with 1g ceftriaxone. While this case was a heterosexual man who reported not having had oral sex, it has been suggested that kissing may be a potential route of transmission to the oropharynx [19]. UK guidelines recommend that anyone with a ceftriaxone-resistant genital infection or infection acquired in the Asia-Pacific region should have pharyngeal sampling performed.

## Conclusions

Early diagnosis, culture and susceptibility testing, contact tracing and test-of-cure remain important to contain spread of ceftriaxone-resistant gonorrhoea. UKHSA has established a molecular assay to detect the *penA*-60 allele and this may be used to aid investigation of treatment failure and outbreaks. Additionally, UKHSA is working to raise awareness of resistant gonorrhoea among people travelling to and from the Asia-Pacific region and among students from this region, including advice on how to access UK sexual health services. Urgent global action is required to stop untreatable gonorrhoea becoming a reality in the near future.
