# Author's correction for Euro Surveill. 2022;27(46)

**DOI:** 10.2807/1560-7917.ES.2022.27.47.221124c

**Published:** 2022-11-24

**Authors:** Eurosurveillance editorial team

**Affiliations:** 1 European Centre for Disease Prevention and Control (ECDC), Stockholm, Sweden

In the article ‘Detection of 10 cases of ceftriaxone-resistant *Neisseria gonorrhoeae* in the UK, December 2021 to June 2022’ by Day et al., published on 17 November 2022, the affiliations of authors Jacqueline Jones and Preneshni Naicker were updated at the request of the authors on 21 November 2022.

